# How to Minimize Light–Organic Matter Interactions for All-Optical Sub-Cutaneous Temperature Sensing

**DOI:** 10.1021/acsomega.1c02057

**Published:** 2021-07-16

**Authors:** Ernesta Heinrich, Yuri Avlasevich, Katharina Landfester, Stanislav Baluschev

**Affiliations:** † Max-Planck-Institute for Polymer Research, Ackermannweg 10, 55128 Mainz, Germany; ‡ Sofia University “St. Kliment Ochridski”, 5 James Bourchier Blvd, 1164 Sofia, Bulgaria

## Abstract

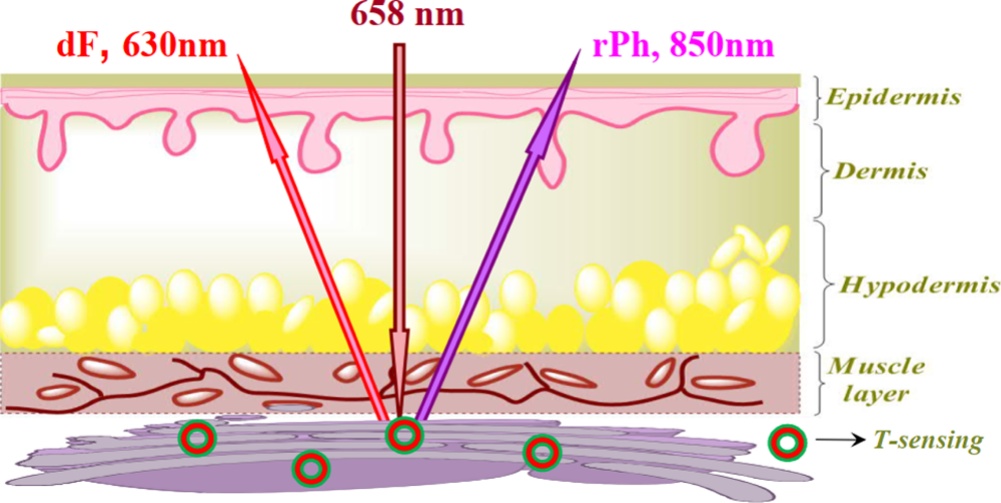

Penetration and emanation of light into tissue are limited by the strong interaction of light with the tissue components, especially oxygenated hemoglobin and white adipose tissue. This limits the possibilities for all-optical minimal invasive sensing. In order to minimize the optical losses of light in and out of the tissue, only a narrow optical window between 630 and 900 nm is available. In this work, we realized for the first time all-optical temperature sensing within the narrow optical window for tissue by using the process of triplet–triplet annihilation photon energy upconversion (TTA-UC) as a sensing tool. For this, we apply the asymmetrical benzo-fused BODIPY dye as an optimal emitter and mixed palladium benzo-naphtho-porphyrins as an optimal sensitizer. The TTA-UC sensing system is excited with λ = 658 nm with an extremely low intensity of 1 mW × cm^–2^ and is factual-protected for a time period longer than 100 s against oxygen-stimulated damage, allowing a stable demonstration of this T-sensing system also in an oxygen-rich environment without losing sensitivity. The sensing dyes we embed in the natural wax/natural matrix, which is intrinsically biocompatible, are approved by the FDA as food additives. The demonstrated temperature sensitivity is higher than Δ*T* = 200 mK placed around the physiologically relevant temperature of *T* = 36 °C.

## Introduction

The many biochemical reactions responsible for cellular functions, which are either exothermic or endothermic, are fundamentally co-regulated by the intracellular temperature distribution. In addition, they are exposed to different oxygen conditions depending on the particular areas within cell organelles, at which they take place.^1,2^ In an ideal case, the minimally invasive thermometry could be used to probe many functional characteristics of biological specimens, their physiological behavior under various conditions, and their responses to external stimuli, such as chemical and environmental stress.^3^ A series of compendious reviews have discussed the progress in biocompatible temperature measurements. Optical methods^4^ for temperature sensing are less invasive and able to provide a time-resolved and two-dimensional spatial evolution of the temperature distribution of a living cell.^5,6^

Optically excited chromophores in the triplet state can be used for applications in various fields, like bioimaging,^7^ molecular sensing,^8^ and photocatalytic organic reactions.^9^ The process of triplet–triplet annihilation photon energy upconversion (TTA-UC) demonstrates good prospects for temperature-sensing applications based on optically excited triplet ensembles. This all-optical sensing technique, supported by ratiometric-type signal registration, ensures relative independence of the data obtained on small excitation intensity instabilities, local molecular concentration variations, and field-of-view uncertainties for the temperature region centered at the physiologically important temperature of 36 °C.^10^

**Figure 1:**
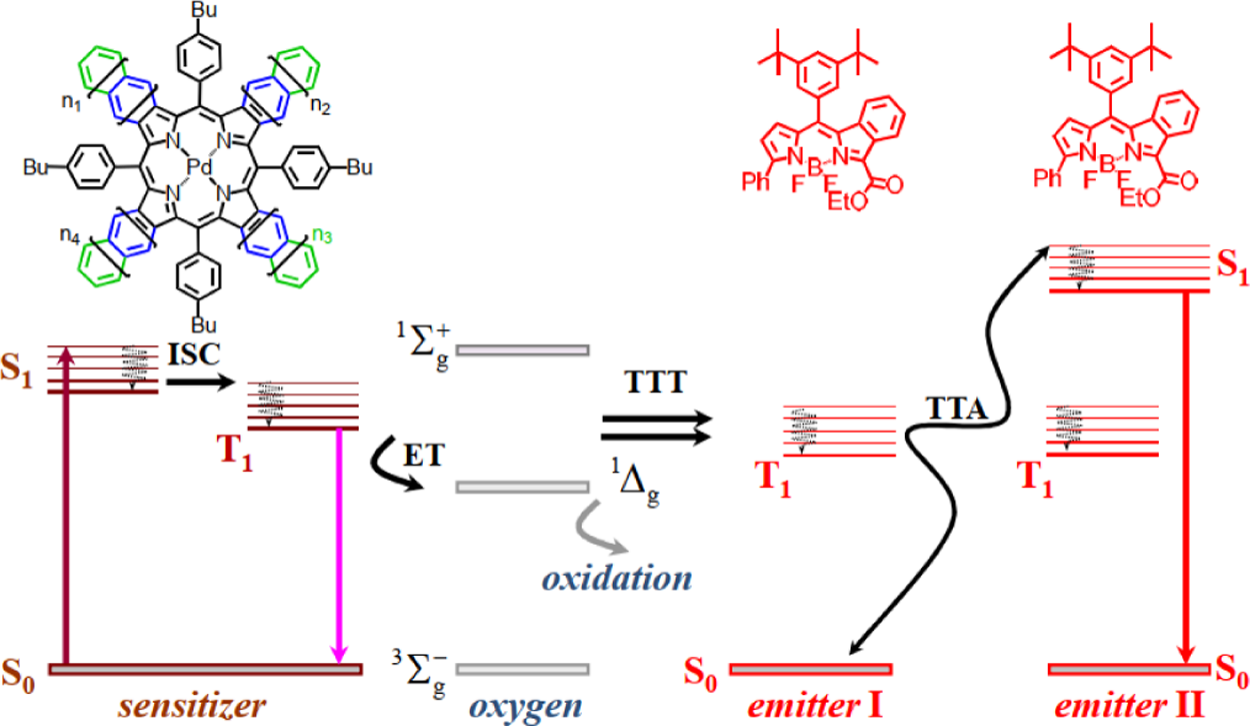
Simplified energetic scheme of the triplet–triplet annihilation upconversion process in an oxygen-rich environment. Inset: chemical structures of the sensitizer-mixed palladium benzo-naphtho-porphyrins, *n* = 1,0 (PdBNP); emitter—MPh-MB-BODIPY.

Briefly, the TTA-UC process is performed in a multi-chromophore system built of energetically optimized pairs of sensitizers (metallated macrocycles) and emitter molecules (aromatic hydrocarbons), as shown in Figure 1.^10^ Photon energy absorbed by the sensitizer (dark red arrow, Figure 1) is stored into the triplet state, created during the process of intersystem crossing (ISC). As a next step, stored energy is transferred to an emitter triplet state via the process of triplet–triplet transfer. Furthermore, the excited triplet states of two emitter molecules go through the triplet–triplet annihilation (TTA) process: so, one emitter molecule relaxes to its singlet ground state, but the other molecule gains the energy of both triplet states and populate the excited emitter singlet state. After radiative relaxation of the emitter singlet state to the ground state, a delayed emitter fluorescence (red arrow, Figure 1, called shortly **dF**), bearing higher energy than that of the excitation photon, is emitted. If triplet manifolds of the emitter and sensitizer molecules are not optimally overlapped or if the molecular rotational diffusion of the interacting sensitizer/emitter triplet moieties is not high enough, complete depopulation of the sensitizer triplet state does not happen: simultaneously, a residual sensitizer phosphorescence (violet arrow, Figure 1, called shortly **rPh**) will be observed.^11,12^

The efficiency and sustainable operation of the TTA-UC process depend drastically on the presence of oxygen molecules, known as effective quenchers of the excited triplet states. Blends of natural waxes/oils with pronounced singlet oxygen scavenging properties, containing TTA-UC molecules, allow for almost complete chemically binding of the locally dissolved molecular oxygen.^13^ Thus, during the excitation, the optically assessed spot is almost oxygen free, and the temperature-sensing procedure can be performed in a sustainable manner. Employing a matrix consisting of natural waxes/oils ensures simultaneously the ability to tune the temperature-sensitivity range toward the biologically relevant temperature window (centered at *T* = 36 °C) and to use natural biocompatible materials (all used waxes/oils are approved from FDA as food additives).

Despite the demonstrated experimental progress of the TTA-UC process as an all-optical sensing tool, efficiently protected against the influence of the local oxygen concentration on the provided temperature data—there is a significant problem preventing straightforward application of the TTA-UC sensing technology *in vitro*: the absorption and scattering properties of the human skin.

**Figure 2:**
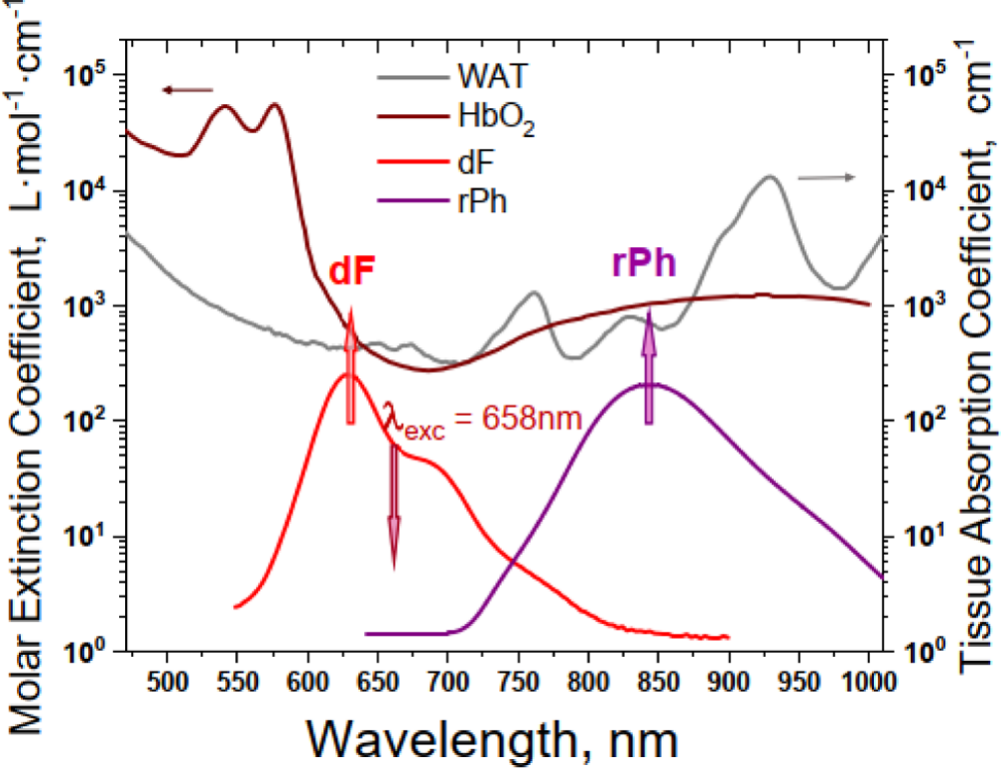
Molar extinction coefficient for different breast tissue components as follows: oxygenated hemoglobin (HbO_2_, dark red line, in water) and purified WAT (gray line) compared with the emission spectral range of the signals of delayed emitter fluorescence (**dF**, the red line) and residual sensitizer phosphorescence (**rPh**, the violet line) excited in the upconversion regime, using deep-red excitation light with an extremely low excitation intensity of 1 mW × cm^–2^.

There is a broad consensus^6−8^ that the optical parameters, as optical absorption and scattering of the living tissue of a particular person, are subject to variations in the blood content, water content, and collagen content, and the fiber development. In order to keep the electromagnetic stress of the patient skin on an acceptable level and to be minimally invasive, the targeted UC-sensing materials must fulfill a chain of very specific requirements: (1) the living organisms develop and accommodate to light intensities close to 1 Sun; therefore, the excitation intensity of the TTA-UC process must be comparable with it; (2) only excitation wavelengths, which coincide with the transparency window of the different components of the human skin penetrate optimally; and (3) simultaneously, in order to keep optical losses low, the emission wavelengths of the optical signals must coincide with the tissue transparency window. Figure 2 demonstrates the optical properties of two components of the human skin, for which absorption spectra are mostly limiting the optical access: oxygenated hemoglobin (HbO_2_, the red curve) and purified white adipose tissue (WAT, the gray curve).

All these requirements predetermine a new, non-orthodox optimization strategy for the process of TTA-UC: until now, all synthetic efforts^14^ were directed toward as possible high anti-Stokes shift of the UC-delayed fluorescence signal. The anti-Stokes shift of the signal of delayed fluorescence is Δ*E*^aS^ ∼ 0.55–0.7 eV. In this respect, in order to squeeze the complete TTA-UC spectrum into the limited human skin transparency window, it is essential to minimize the anti-Stokes shift (the studied TTA-UC system demonstrates at least four times smaller Δ*E*^aS^ ∼ 0.08–0.15 eV). The UC-fluorescence signal with central emission wavelength λ ≤ 620 nm is strongly absorbed (Figure 2, please refer to the HbO_2_—absorption). Even, if such a delayed fluorescence signal is generated into the studied tissue, only a small part of this emission will be able to escape out. Similarly, if the central emission wavelength of the residual sensitizer phosphorescence is λ ≥ 900 nm (please refer to WAT-absorption/optical scattering, Figure 2), the phosphorescence signal experiences similar problems.

## Results and Discussion

Efficient TTA-UC was demonstrated with various sensitizer molecules; in most cases, these were Pd-porphyrins, while simple octaethyl- and tetraphenylporphyrins show Q-band absorption in the green region,^17^ benzoannulated porphyrins (benzo-,^18^ naphtha-,^19^ and anthra-^20^) have a Q-band absorption in red, deep-red, and IR-A region, respectively. However, a symmetric benzoannulation on all four positions of a porphyrin ring leads to a drastic bathochromic shift of the absorption (80–100 nm), stepwise annulation of one, two, or three benzene moieties allows small shifts of Q-band absorption in order of 20–30 nm.^15^ It was demonstrated that such asymmetric porphyrins act as efficient sensitizers in TTA-UC.^18,19^ A similar synthetic strategy was applied for the UC emitters: for each sensitizer, a suitable emitter with the highest UC efficiency could be prepared by modification of the π-core of anthracene,^21^ tetracene,^22^ perylene,^23^ or BODIPY^24^ dyes.

In the present paper, we combined a mixed pyrrole condensation strategy, previously known for porphyrins^15,19^ with benzo-annulation on a pyrrole ring, for the synthesis of new core-modified BODIPY dye having a high fluorescence quantum yield, good photochemical stability, and acting as an efficient singlet emitter in the TTA-UC process with mixed palladium benzo-naphtho-porphyrins as a sensitizer. BODIPY was chosen on purpose because the energy position of the triplet state^25^ is laying relatively high; thus, the anti-Stokes shift of the resulting UC-emission was expected to be low.

Modification of BODIPY dyes via π-extension is a known method to shift their absorption bathochromically.^25^ Introduction of a phenyl ring is a common way to make monofunctional dyes, whereas a substitution of pyrrole with aryl groups at the alpha position shifts the absorption significantly.^26^ Another way is the benzoannulation,^27^ similar to porphyrins and perylene dyes.^28^ For the double annulation, the same synthetic precursors as for tetrabenzoporphyrins can be used. Recently, monobenzo-BODIPY was prepared by a reaction with tetrahydroisoindole with formylpyrrole.^29^

**Scheme 1:**
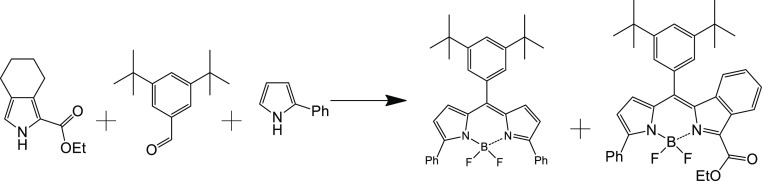
Synthesis of DPh-BODIPY and MPh-MB-BODIPY

**Scheme 2:**
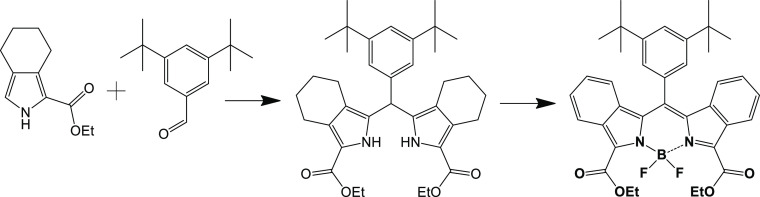
Synthesis of DB-BODIPY

Here, we used two pyrroles and one aldehyde to obtain a statistical acid-mediated condensation with subsequent separation of the products by column chromatography (Scheme 1). In the first step, trifluoroacetic acid (TFA) was used as a catalyst to afford dipyrromethane intermediates, which were oxidized under mild conditions to the corresponding dipyrromethenes. Then, the reaction with boron trifluorate etherate afforded BODIPY dyes. The first one, 3,5-diphenyl-8-(3,5-di-*tert*-butylphenyl)BODIPY (DPh-BODIPY) could be isolated directly after this step, but we used the mixture for the final aromatization procedure, which was performed with 2,3-dichloro-5,6-dicyano-1,4-benzoquinone (DDQ) in toluene under reflux. Surprisingly, only DPh-BODIPY and 3-phenyl-5-ethoxycarbonyl-6:7-benzo-8-(3,5-di-*tert*-butylphenyl) BODIPY (MPh-MB-BODIPY) were separated after column chromatography. No evidence for the formation of 3,5-bis(ethoxycarbonyl)-1,2,6,7-dibenzo-8-(3,5-di-*tert*-butylphenyl)BODIPY (DB-BODIPY) or its non-oxidized precursors was found. For the synthesis of DB-BODIPY, only aldehyde and tetrahydroisoindole ester were used. After similar steps, DB-BODIPY was isolated as a blue solid (Scheme 2). The obtained three BODIPY dyes are strongly colored solids; their solutions show a ranging from deep-red to blue colors. Strong fluorescence of first two dyes was visible even by the naked eye. Absorption spectra revealed that every annulation step shifts the absorption bathochromically by 40–45 nm; at the same time, molar absorptivity is growing as well, due to enlargement of the π-system. All dyes show bright fluorescence, with quantum yields of 0.56–0.74 and a Stokes shift of 608–1033 cm^–1^ (see Table 1).

**Table 1:** Spectral Properties of BODIPYs in Toluene at Room Temperature

	absorbance λ_max_ [nm]	ε [M^–1^ cm^–1^]	emission λ_max_ [nm]	Φ_f_^a^	Stokes shift [cm^–1^]
DPh-BODIPY	557	30,600	591	0.68	1033
MPh-MB-BODIPY	597	52,650	630	0.74	877
DB-BODIPY	641	67,100	667	0.56	608

a Fluorescence quantum yields for all BODIPYs (λ_exc_ = 560 nm) were calculated using Lumogen Red as a standard (Φ_f_ = 0.96 in chloroform).

**Figure 3:**
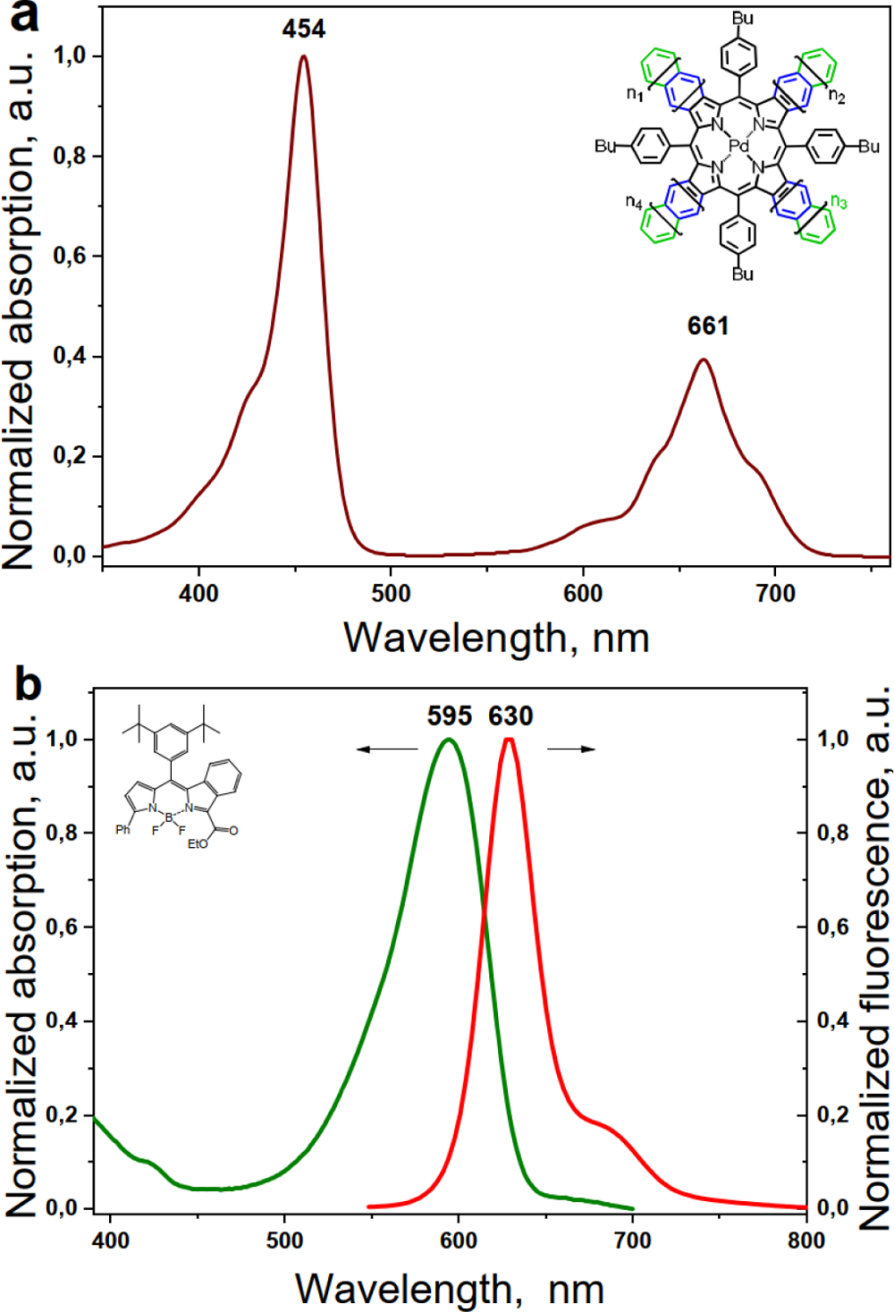
(a) Normalized absorption spectrum of the mixed palladium benzo-naphtho-porphyrin family (PdBNP); (b) normalized absorption (the green curve) and fluorescence (the red curve) of MPh-MB-BODIPY in toluene.

The normalized absorption and fluorescence spectra of the asymmetric BODIPY, together with the normalized absorption spectrum of the family of mixed benzo-naphtho-porphyrins are shown in Figure 3. The absorption and fluorescence spectra of the symmetric BODIPYs—the DPh-BODIPY and the DB-BODIPY—are shown in the Supporting Information, Figures S4 and S5, respectively. As expected^24b^ the studied BODIPY’s demonstrate efficient TTA-UC when the UC-couples are combined, as shown in Table 2. As seen from the Table 2, the UC system with the smallest anti-Stokes shift is the system obtained by the mixed-condensation strategy, both for the sensitizer and for the emitter molecules. Despite this advantage, there are three other optical parameters derived from Figure 2 and Table 2 making the UC dye-couple PdBNP/MPh-MB-BODIPY an optimal system for under-cutaneous sensing applications: (1) comparing the absorption coefficients of the HbO_2_ for the specific excitation wavelengths λ = 635 nm (the UC-couple PdTBP/DPh-BODIPY) and λ = 658 nm (the UC-couple PdBNP/MPh-MB-BODIPY), there is a more than a 1.5 times higher absorption for the shorter excitation wavelength; (2) the crucial advantage of the asymmetrical UC-couple is the fact that the emission generated inside the tissue will be more than 5.3 times less absorbed than the signal of the UC dye-couple PdTBP/DPh-BODIPY; and (3) regarding the absorption coefficient for the residual phosphorescence signal, the asymmetrical UC dye-couple reveals more than 7.5 times lower optical losses than it is observed for the strongly red-shifted UC dye-couple PdTNP/DB-BODIPY.

**Table 2:** TTA—UC Parameters for Different UC-Couples, in Toluene at Room Temperature, Glovebox Conditions

sensitizer	emitter	excitation [nm]	**dF** λ_max_ [nm]	**rPh** λ_max_ [nm]	anti-Stokes shift [cm^–1^]	Q.Y. TTA-UC
PdTBP	DPh-BODIPY	635	591	795	1170 (0.145 eV)	0.02
PdBNP	MPh-MB-BODIPY	658	630	850	677 (0.084 eV)	0.021
PdTNP	DB-BODIPY	705	667	900	806 (0.100 eV)	0.018

Summarizing the data presented in (1), (2), and (3), one can conclude that the registered **dF** or **rPh** signals for the UC dye-couple PdBNP/MPh-MB-BODIPY collected after the sequential processes—excitation (tissue penetration), TTA-UC, that is, generation of delayed fluorescence and residual phosphorescence, emanation of the optical signal (escape from the tissue)—are more than eight times higher, keeping all other experimental conditions the same (namely, excitation photon flux, TTA-UC quantum yield, dye concentrations, oxygen content, sample temperature, etc.) constant.

**Figure 4:**
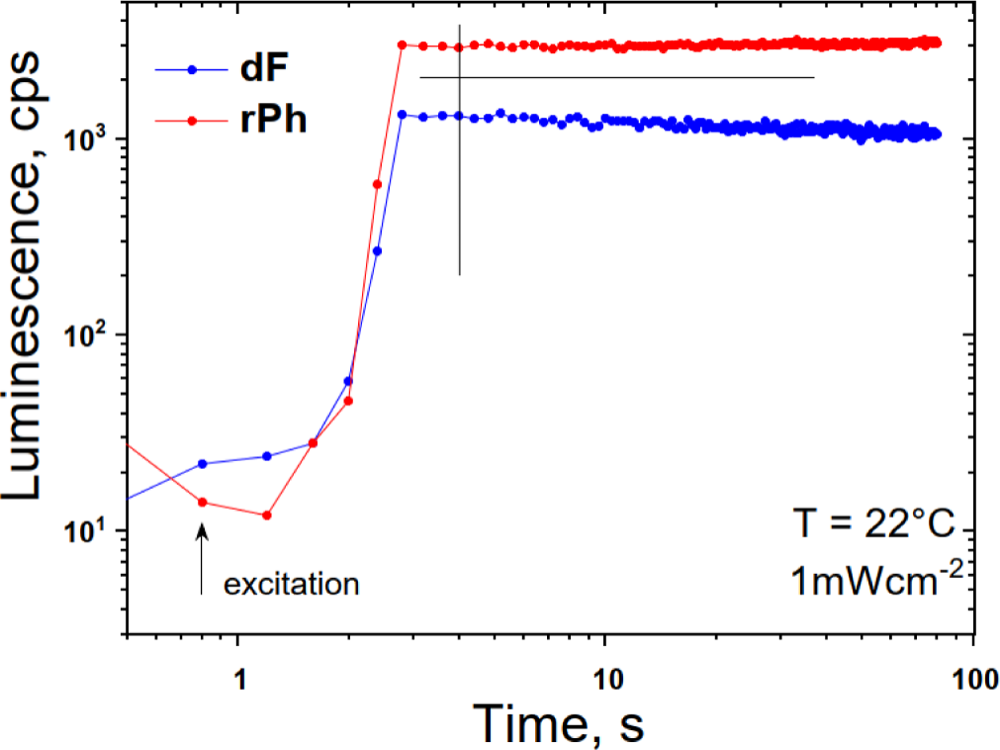
Temporal evolution of the signals of **dF** and **rPh** at sample temperature of *T* = 22 °C. The excitation intensity is kept constant at 1 mW × cm^–2^ for all measurements; cw—diode laser at λ_exc_ = 658 nm; air-saturated environment; excitation spot diameter *d* = 1.8 × 10^–3^ m; sample thickness *b* = 4 × 10^–4^ m. Material composition, as follows, 1 × 10^–5^ M PdBNP/2 × 10^–4^ M MPh-MB-BODIPY/40 wt % carnauba wax/30 wt % squalene oil/30 wt % peanut oil. The black lines are guide for the eye.

In an oxygen-contaminated environment, during the optical excitation, singlet oxygen is generated continuously. The phytochemical compounds of the vegetable oils (e.g., tocopherol, tocotrienol, and γ-oryzanol) demonstrate a remarkable ability to bind chemically all existing amounts of singlet oxygen. If the oxygen permeation rate through the sample surface is much lower than the rate of chemical binding of singlet oxygen across the optically assessed spot, after a short initial period (around 4 s in this case, see Figure 4), the entire oxygen content is chemically bound. This fact is demonstrated by the truly stationary intensity of the signals of **dF** and **rPh**, as verified in Figure 4.

**Figure 5:**
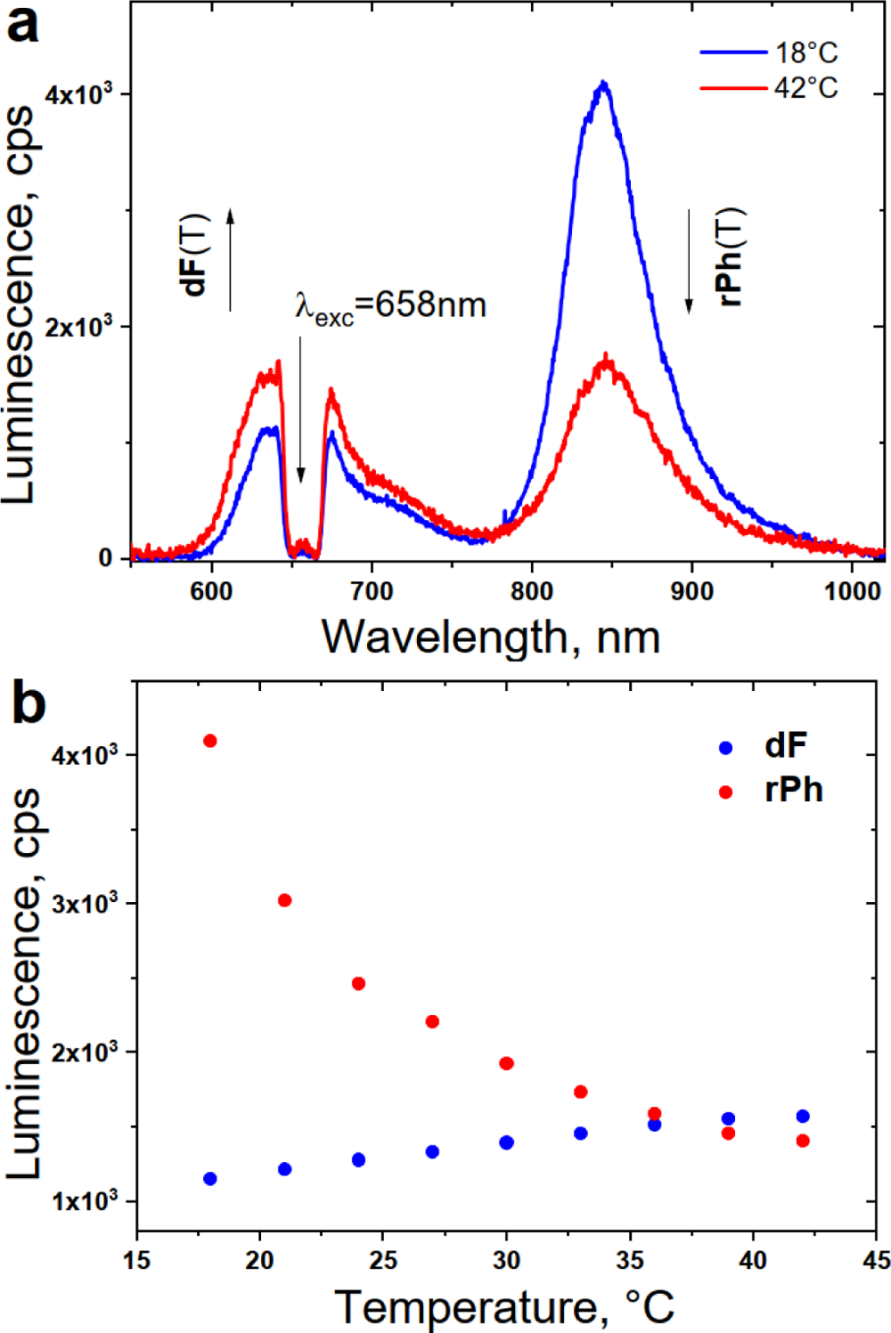
(a) Luminescence spectra of the UC systems for different sample temperatures; (b) temperature dependence of the signals of **dF** (at λ_max_ = 630 nm, the blue dots) and **rPh** (at λ_max_ = 850 nm, the red dots) on the sample temperature. Experimental conditions for all measurements: material composition, as follows, 1 × 10^–5^ M PdBNP/2 × 10^–4^ M MPh-MB-BODIPY/40 wt % carnauba wax/30 wt % squalene/30 wt % peanut oil. The spectra are obtained at the *t* = 4 s after starting the optical excitation. The excitation intensity is kept constant, at 1 mW × cm^–2^ for all measurements; *cw*—diode laser at λ_exc_ = 658 nm; air saturated environment.

The signals of **dF** and **rPh**, even in an oxygen saturated environment, demonstrate remarkable stability. This allows us to study the temperature dependence of the TTA-UC process. In Figure 5a, the luminescence spectra of the studied material composition are demonstrated for two boundary temperature values, namely, 18 and 42 °C. As expected,^10^ a significant decrease in the residual sensitizer phosphorescence, accompanied with a well-observable increase in the emitter delayed fluorescence with increasing sample temperature, was detected. The data presented in Figure 5a are summarized in Figure 5b, where the dependence of the **dF** and **rPh** signals for a stepwise increase in the sample temperature is reported.

**Figure 6:**
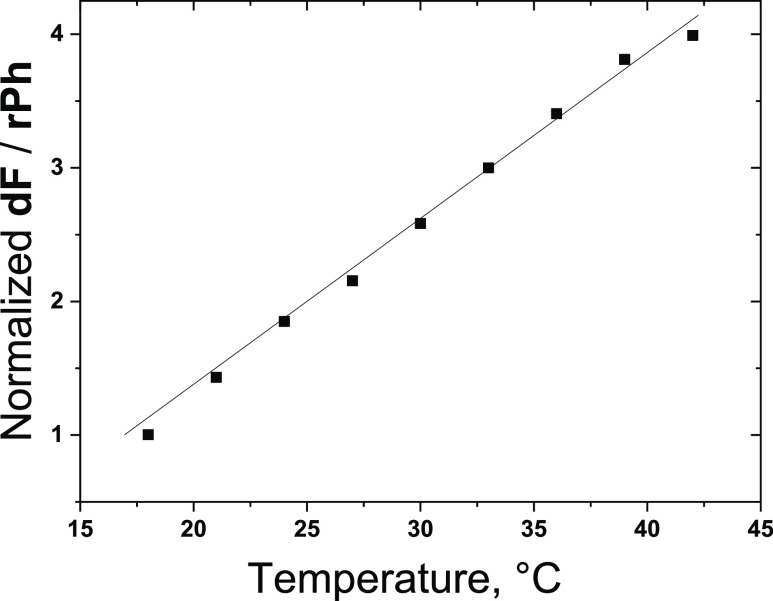
Temperature calibration curve—ratiometric response. Normalized ratio of the signals of **dF/rPh** as a function of the sample temperature, as demonstrated in Figure 5b.

As shown in Figure 5b, the signals of **dF** and **rPh** have comparable intensity. Additionally, the **dF** signal increases monotonically with increasing sample temperature; simultaneously, the **rPh** signal decreases monotonically with increasing sample temperature. Thus, it allows us to achieve a non-ambiguous calibration curve, as shown in Figure 6. From this figure, it is evident that this biocompatible material composition (1 × 10^–5^ M PdBNP/2 × 10^–4^ M MPh-MB-BODIPY/40 wt % carnauba wax/30 wt % squalene oil/30 wt % peanut oil) demonstrates a high-temperature sensitivity since the ratio **dF**/**rPh** is changed more than four times within the physiologically relevant temperature window of interest Δ*T* ∼ 18 – 42 °C.

## Conclusions

In this work, we demonstrated for the first time all-optical temperature sensing with optimal excitation/emanation of the optical signals. The synthesized asymmetrical benzo-fused BODIPY was identified as an optimal emitter for the process of TTA-UC, performed with the mixed palladium benzo-naphtho-porphyrins, used as a sensitizer. The sensing technique is based on a ratiometric-type signal registration that ensures significant independence of the obtained data on excitation intensity instabilities, local molecular concentration fluctuations and field-of-view variations. The identified that matrix materials (natural wax/natural oils) are inherently biocompatible and FDA-approved as food additives. The desired temperature sensitivity is better than 200 mK centered on the physiologically relevant temperature of 36 °C and is warranted by using the process of TTA-UC as a sensing mechanism. The TTA-UC system is effectively protected for more than 100 s against oxygen-induced damages, allowing stable performance of this temperature-sensing system even in the ambient environment without losing sensitivity while applying the same calibration curve.

## Experimental Section

3,5-Di(*tert*-butyl)benzaldehyde (TCI Chemicals), DDQ, carnauba wax, squalene, peanut oil (Acros), triethylamine (Roth), DIPEA (Roth), boron trifluoride etherate (Merck), anhydrous dichloromethane (Aldrich), and 2-phenylpyrrole (Chempur) were used as received. Ethyl-4,5,6,7-tetrahydro-2*H*-isoindole-1-carboxylate was synthesized, as described elsewhere.^15^^1^H and ^13^C NMR spectra were recorded on a Bruker Avance 250 and a Bruker Avance 500 spectrometers. Chemical shifts are denoted in d unit (ppm). Mass spectra were recorded with an Advion Expression L spectrometer. UV/Vis spectra were recorded at room temperature on a Shimadzu UV-1800 spectrophotometer. Fluorescence spectra were recorded on a Spex Fluorolog 3 spectrometer. Fluorescence quantum yields were determined using the relative method using Lumogen Red as a ref (16).

### Synthesis of DPh-BODIPY and MPh-MB-BODIPY

3,5-Di(*tert*-butyl)benzaldehyde (218 mg, 1 mmol), ethyl-4,5,6,7-tetrahydro-2*H*-isoindole-1-carboxylate (193 mg, 1 mmol), and 2-phenylpyrrole (143 mg, 1 mmol) were dissolved in 100 mL of absolute CH_2_Cl_2_ under an Ar atmosphere. Three drops of TFA were added, and the solution was stirred at room temperature overnight in the darkness. Dry DDQ (250 mg) was added and stirring was continued for 2 h. Triethylamine (2 mL) was added, and the organic phase was washed with aqueous sodium sulfite (3%, 2 × 100 mL). Organic layers were separated, dried over anhydrous sodium sulfate, and evaporated to dryness. *N,N*-diisopropylethylamine (DIEA) (3 mL) and 100 mL of absolute CH_2_Cl_2_ were added under an Ar atmosphere, and the solution was stirred at room temperature for 10 min. BF_3_·OEt_2_ (3 mL) was added, and stirring was continued for 2 h. The reaction mixture was washed with NaHCO_3_ solution (5%, 2 × 100 mL) and water (100 mL). The combined organic extracts were dried over Na_2_SO_4_, filtered, and evaporated. Toluene (50 mL) and 1,4-dioxane (100 mL) were added, stirred for 5 min, and then, DDQ (300 mg) was added and stirred at 110 °C for 15 h. Solution was cooled and washed with aqueous sodium sulfite (3%, 2 × 100 mL). Organic layers were separated, dried over anhydrous sodium sulfate, and evaporated to dryness. Column chromatography with silica gel (eluent–toluene) afforded DPh-BODIPY as a first red fraction with yellow fluorescence, which was evaporated and recrystallized from CH_2_Cl_2_/methanol to afford dark red crystals after drying under vacuum. Yield 207 mg (39%).

^1^H NMR (250 MHz, C_2_D_2_Cl_4_): δ 7.91–7.87 (m, 4H), 7.63 (s, 1H), 7.49–7.45 (m, 8H), 6.99 (d, *J* = 4.3 Hz, 1H), 6.67 (d, *J* = 4.2 Hz, 1H), 1.42 (s, 18H). ^13^C NMR (126 MHz, C_2_D_2_Cl_4_): δ 158.12, 150.70, 146.04, 136.35, 133.22, 132.65, 125.25, 124.30, 120.91, 74.13, 34.83, 31.39; λ_max_ (toluene)/nm 557 (ε/dm^3^ mol^–1^ cm^–1^ 30,600); fluorescence (toluene): λ_max_ = 591 nm (ϕ = 68%); MS (FD, 8 kV): *m*/*z* (%) 532.5 (100), M^+^; the second violet fraction, possessing red fluorescence, was evaporated, dissolved in cyclohexane (20 mL) and freeze-dried for 24 h, to afford MPhMB-BODIPY as a violet powder (133 mg, 23% yield).

^1^H NMR (250 MHz, C_2_D_2_Cl_4_): δ 8.05 (d, *J* = 8.1 Hz, 1H), 7.99–7.96 (m, 2H), 7,68 (s, 1H), 7.53–7.51 (m, 3H), 7.38–7.36 (m, 2H), 7.31 (t, *J* = 7.6 Hz, 1H), 7.16 (t, *J* = 7.6 Hz, 1H), 6.84 (d, *J* = 4.3 Hz, 1H), 6.67 (d, *J* = 4.2 Hz, 1H), 6.40 (d, *J* = 8.4 Hz, 1H), 4.56 (q, *J* = 7.1 Hz, 2H), 1.51 (t, *J* = 7.1 Hz, 3H), 1.39 (s, 18H). ^13^C NMR (126 MHz, C_2_D_2_Cl_4_): δ 160.50, 158.10, 151.27, 144.28, 139.99, 137.60, 134.62, 132.53, 132.29, 130.68, 130.46, 129.38, 128.28, 123.84, 123.45, 121.87, 74.04, 62.25, 34.93, 31.31, 29.60, 14.13; λ_max_ (toluene)/nm 597 (ε/dm^3^ mol^–1^ cm^–1^ 52,650); fluorescence (toluene): λ_max_ = 630 nm (ϕ = 74%); MS (FD, 8 kV): *m*/*z* (%) 578.6 (100), M^+^; DPh-BODIPY was also synthesized directly from 3,5-di(*tert*-butyl)benzaldehyde and 2-phenylpyrrole following the same procedure but without last aromatization step. The analytical data are identical to those, obtained by mixed pyrrole condensation. Yield 57%.

### Synthesis of DB-BODIPY

3,5-Di(*tert*-butyl)benzaldehyde (218 mg, 1 mmol) and ethyl-4,5,6,7-tetrahydro-2H-isoindole-1-carboxylate (386 mg, 2 mmol) were dissolved in 100 mL of absolute CH_2_Cl_2_ under an Ar atmosphere. Three drops of TFA were added, and the solution was stirred at room temperature overnight in the darkness. Dry DDQ (250 mg) was added, and stirring was continued for 2 h. Triethylamine (2 mL) was added, and the organic phase was washed with aqueous sodium sulfite (3%, 2 × 100 mL). Organic layers were separated, dried over anhydrous sodium sulfate, and evaporated to dryness. DIEA (3 mL) and 100 mL of absolute CH_2_Cl_2_ were added under an Ar atmosphere, and the solution was stirred at room temperature for 10 min. BF_3_·OEt_2_ (3 mL) was added, and stirring was continued for 4 h. The reaction mixture was washed with NaHCO_3_ solution (5%, 2 × 100 mL) and water (100 mL). The combined organic extracts were dried over Na_2_SO_4_, filtered, and evaporated. Toluene (50 mL) and 1,4-dioxane (100 mL) were added, stirred for 5 min, and then, DDQ (500 mg) was added and stirred at 110 °C for 15 h. Solution was cooled and washed with aqueous sodium sulfite (3%, 2 × 100 mL). Organic layers were separated, dried over anhydrous sodium sulfate, and evaporated to dryness. Column chromatography with silica gel (eluent–toluene) afforded blue fraction, which was evaporated, dissolved in cyclohexane, and freeze-dried for 24 h to afford a blue powder. Yield 230 mg (37%).

^1^H NMR (250 MHz, C_2_D_2_Cl_4_): δ 8.09 (d, *J* = 8.2 Hz, 2H), 7,76 (s, 1H), 7.36 (d, *J* = 1.7 Hz, 2H), 7.33–7.25 (m, 2H), 7.19–7.04 (m, 2H), 6.22 (d, *J* = 8.4 Hz, 2H), 4.61 (q, *J* = 7.1 Hz, 4H), 1.55 (t, *J* = 7.1 Hz, 6H), 1.38 (s, 18H). ^13^C NMR (126 MHz, C_2_D_2_Cl_4_): δ 160.45, 152.68, 142.82, 139.62, 134.58, 132.69, 130.78, 129.62, 129.16, 126.60, 123.76, 123.08, 122.57, 121.85, 74.04, 74.00, 73.78, 73.56, 62.27, 35.12, 31.27, 26.82, 14.18; λ_max_ (toluene)/nm 641 (ε/dm^3^ mol^–1^ cm^–1^ 67,100); fluorescence (toluene): λ_max_ = 667 nm (ϕ = 56%); MS (FD, 8 kV): *m*/*z* (%) 624.6 (100), M^+^.

## Supporting Information Available

Solvents and chemicals used were of commercial grade purity; column chromatography performed on silica gel (Geduran Si60, Merck); HPLC carried out using Agilent 1200 setup at room temperature (pump and photodiode array detector; column—Agilent Eclipse Plus C18 L = 100 mm/diameter = 4.6 mm/particle size = 3.5 μm, wavelength = 260 nm, eluent: gradient starting THF/methanol = 5:95 up to 60:40 with 1 mL/min); ^1^H and ^13^C NMR spectra recorded on Bruker DPX 250 and Bruker Avance 300 spectrometers; and FD mass spectra measured with a VG Instruments ZAB 2-SE-FPD instrument (PDF)
