# Supplementary material for: How to Minimize Light–Organic Matter Interactions for All-Optical Sub-Cutaneous Temperature Sensing
Source: ACS Omega. 2021 Jul 16;6(29):18860–7. doi: 10.1021/acsomega.1c02057 (PMC8320075; doi:10.1021/acsomega.1c02057)
Supplement: Supplementary file 1 — ao1c02057_si_001.pdf [file ao1c02057_si_001.pdf]

Supporting Information

How to minimize light – organic matter interaction for all-optical sub-cutaneous temperature sensing

Ernesta Heinrich[1], Yuri Avlasevich[1], Katharina Landfester[1*] and Stanislav Baluschev[1,2*]

[1] Max-Planck-Institute for Polymer Research, Ackermannweg 10, 55128 Mainz, Germany

[2] *Sofia University "St. Kliment Ochridski", 5 James Bourchier Blvd, 1164 Sofia, Bulgaria*

KEYWORDS: minimally invasive temperature sensing; sub-cutaneous; biocompatible; optimal optical excitation and emanation; triplet-triplet annihilation upconversion; asymmetrically π-extended BODIPY.

AUTHOR INFORMATION
Ernesta Heinrich, heinrich.e.2017@gmail.com;
Yuri Avlasevich, avlasevi@mpip-mainz.mpg.de ; https://orcid.org/0000-0002-2320-4976
Katharina Landfester, landfest@mpip-mainz.mpg.de; https://orcid.org/0000-0001-9591-4638
Stanislav Baluschev, balouche@phys.uni-sofia.bg; *https://orcid.org/0000-0002-0742-0687*

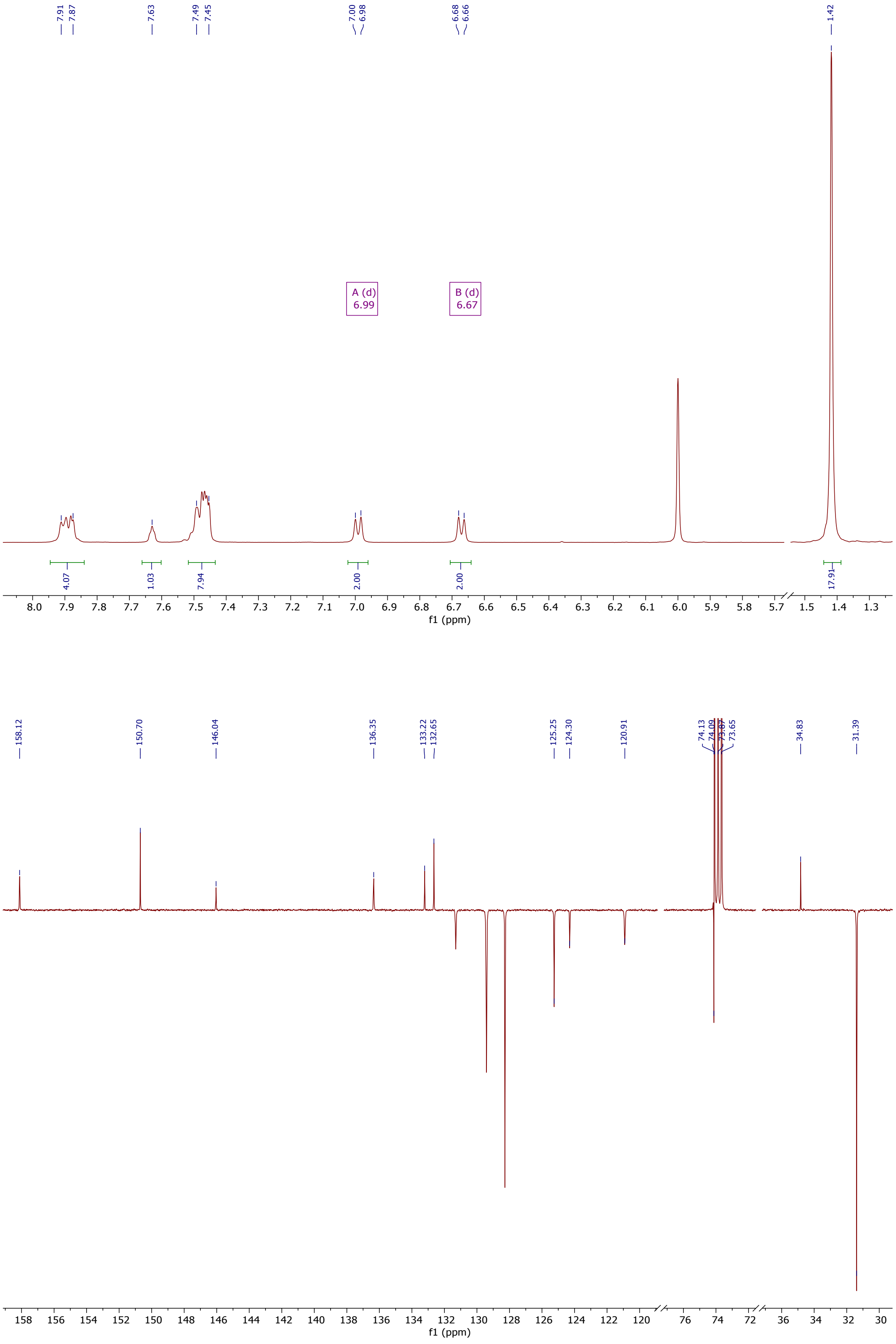

Figure S1. ^{1}H-NMR and ^{13}C-NMR spectra of DPh-BODIPY and MPh-MB-BODIPY in $C_2D_2Cl_4$.

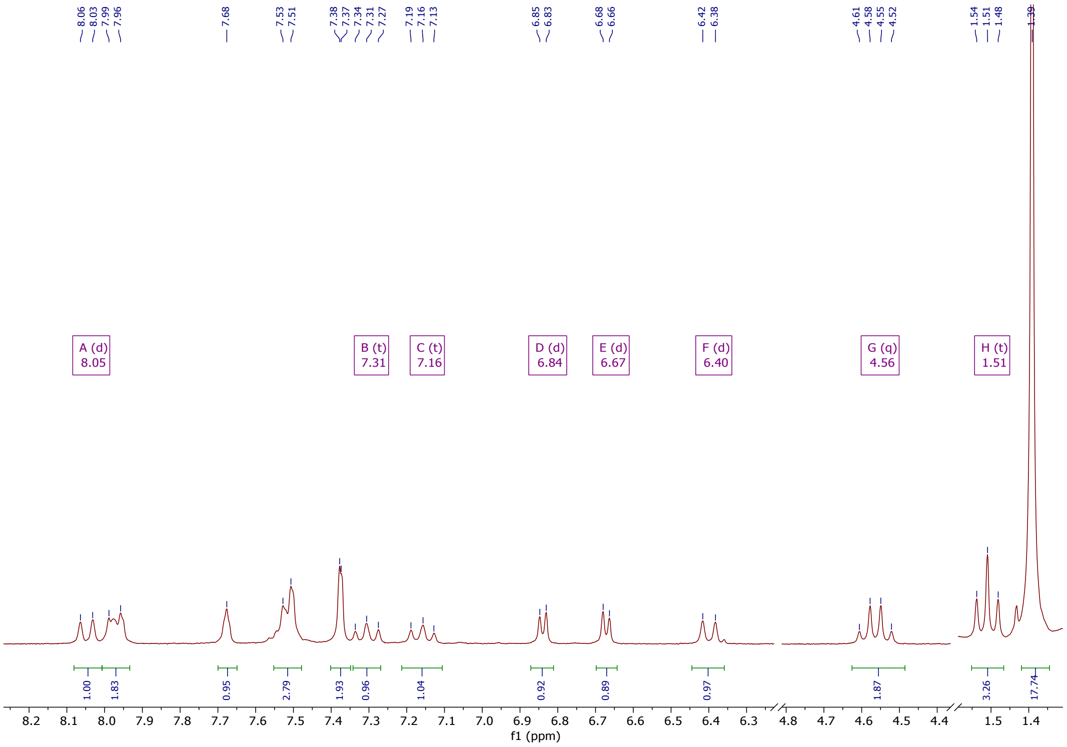

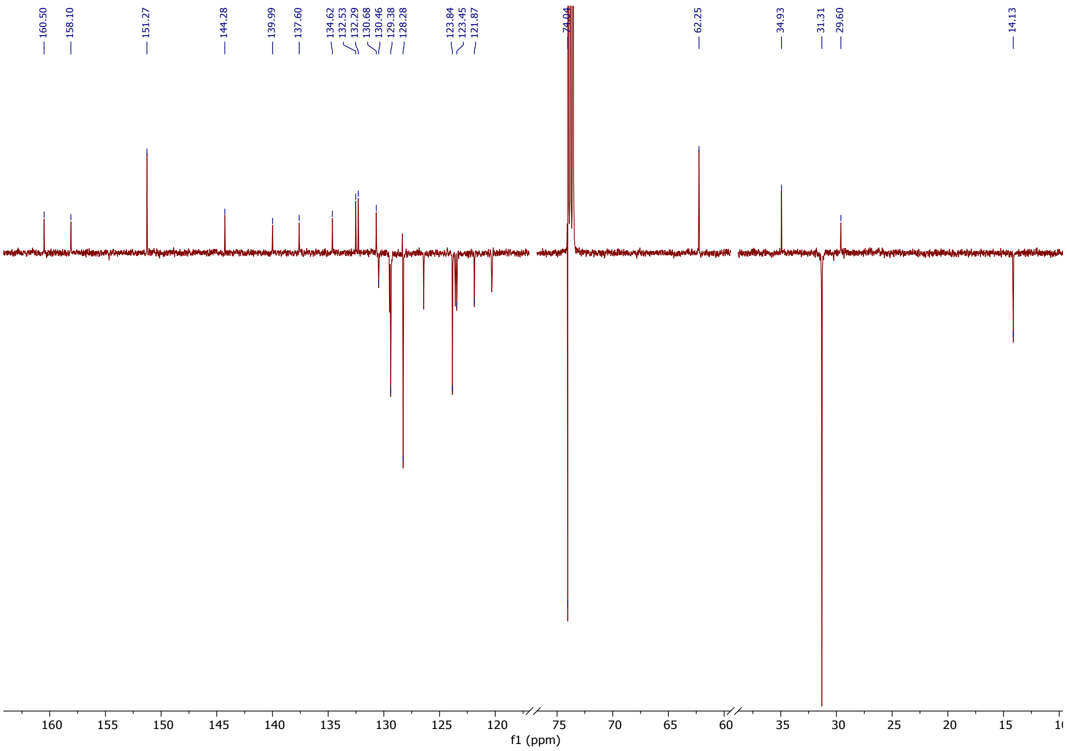

Figure S2. ^{1}H-NMR and ^{13}C-NMR spectra of MPh-MB-BODIPY in $C_2D_2Cl_4$.

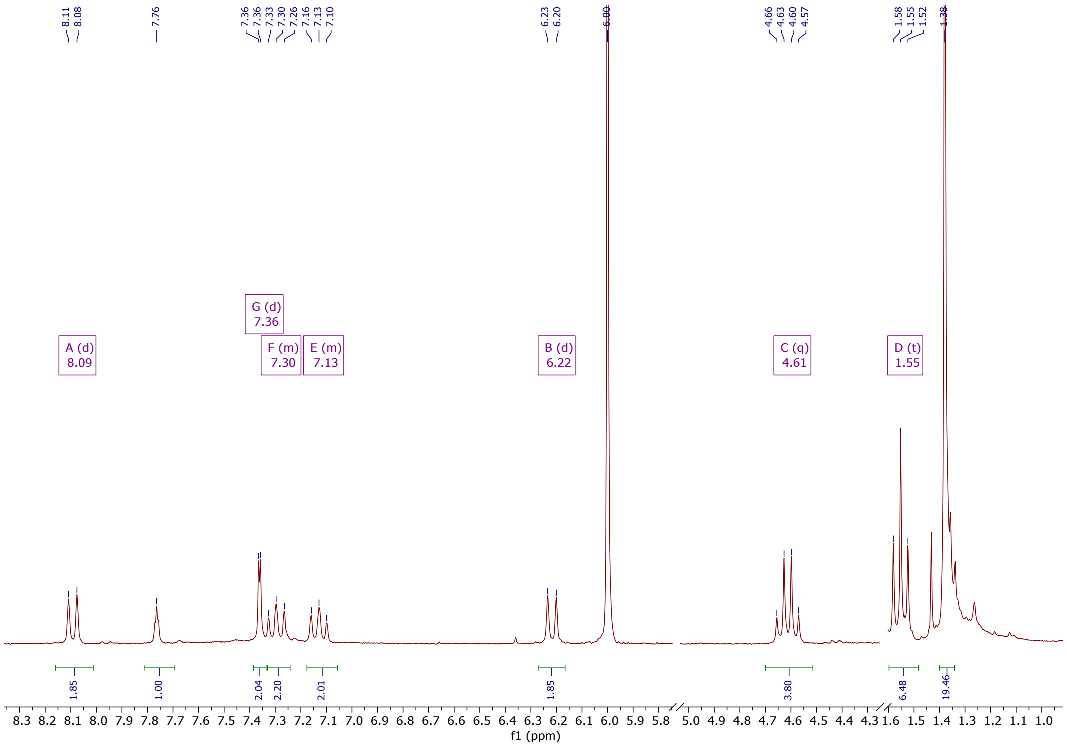

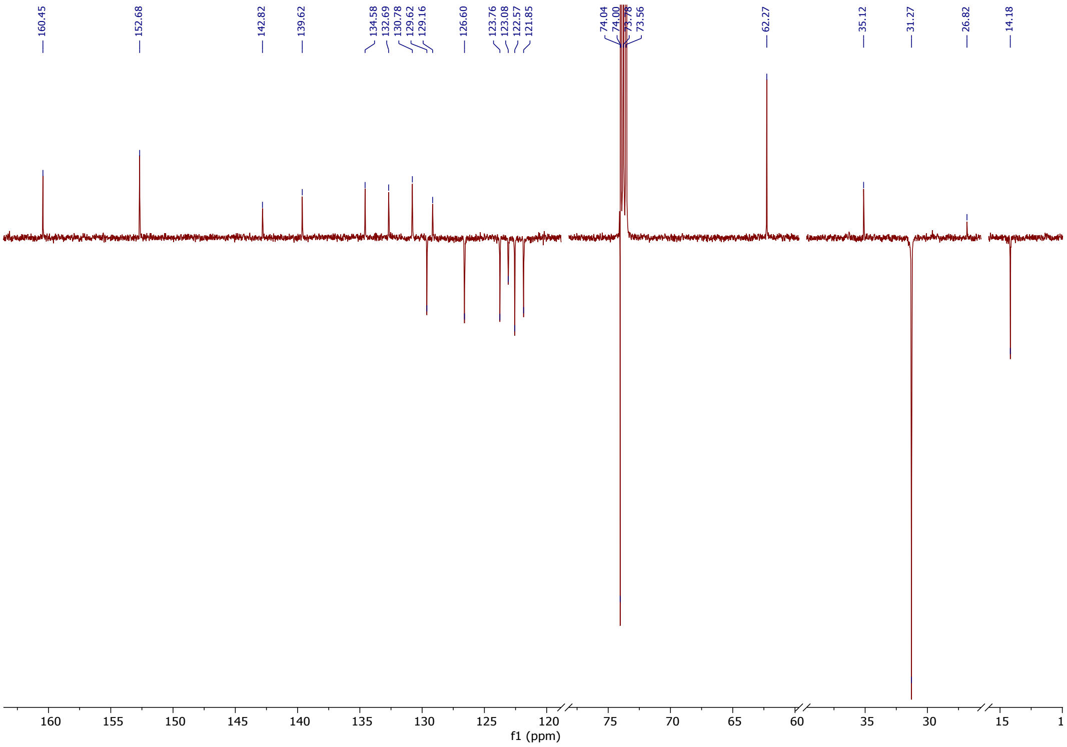

Figure S3. ^{1}H-NMR and ^{13}C-NMR spectra of DB-BODIPY in $C_2D_2Cl_4$.

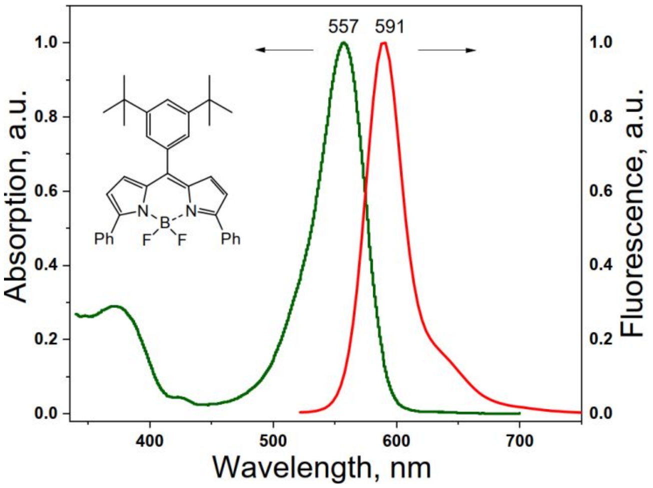

Figure S4: Normalized absorption (the green curve) and normalized fluorescence (the red curve) of DPh- BODIPY in toluene. **Inset**: structure of DPh- BODIPY.

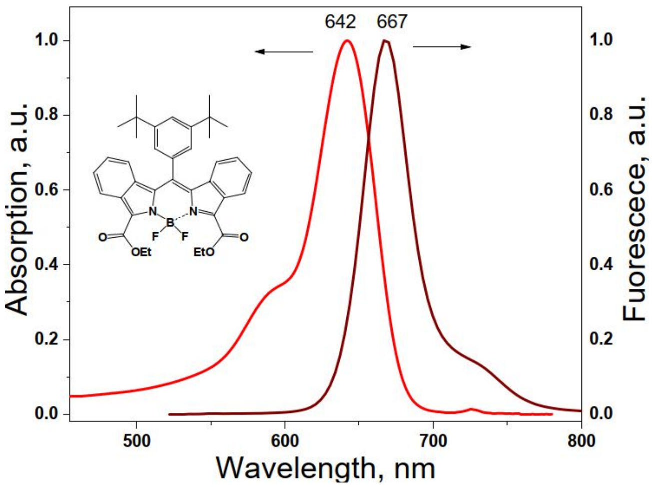

Figure S5: Normalized absorption (the red curve) and fluorescence (the brown curve) of DB-BODIPY in toluene. **Inset**: structure of DB- BODIPY.
